# Historical Changes in the Ecosystem Condition of a Small Mountain Lake over the Past 60 Years as Revealed by Plankton Remains and *Daphnia* Ephippial Carapaces Stored in Lake Sediments

**DOI:** 10.1371/journal.pone.0119767

**Published:** 2015-03-10

**Authors:** Hajime Ohtsuki, Tamotsu Awano, Narumi K. Tsugeki, Seiji Ishida, Hirotaka Oda, Wataru Makino, Jotaro Urabe

**Affiliations:** 1 Graduate School of Life Sciences, Tohoku University, Sendai, Miyagi, Japan; 2 Chronological Research, Nagoya University, Nagoya, Aichi, Japan; National Taiwan Ocean University, TAIWAN

## Abstract

To examine if changes in species composition of a plankton community in the past due to anthropogenic activities can be clarified in lakes without any monitoring data, we analyzed genetically ephippial carapaces of *Daphnia* with plankton remains stored in the bottom sediments of Lake Hataya Ohunma in Japan. In the lake, abundance of most plankton remains in the sediments was limited and TP flux was at low levels (2–4 mg/m^2^/y) before 1970. However TP flux increased two-fold during the period from 1980s to 1990s. In parallel with this increase, abundance of most plankton remains increased although abundance of benthic testate amoebae’s remains decreased, indicating that the lake trophic condition had changed from oligo- to mesotrophic for the past 60 years. According to cluster analysis, the stratigraphic sediments were divided into two periods with different features of the phytoplankton composition. Chronological comparison with events in the watershed suggested that eutrophication occurred because of an increase in visitors to the watershed and deposition of atmospheric dust. In this lake more than 50% of resting eggs produced by *Daphnia* over the past 60 years hatched. However, genetic analysis of the ephippial carapaces (remains) showed that the *Daphnia* population was originally composed of *D*. *dentifera* but that *D*. *galeata*, or its hybrid with *D*. *dentifera*, invaded and increased the population density when the lake was eutrophied. Subsequently, large *D*. *pulex* established populations in the 1980s when largemouth bass were anonymously introduced. These results indicated that the Lake Hataya Ohunma plankton community underwent significant changes despite the fact that there were no notable changes in land cover or land use in the watershed. Since increases in atmospheric deposition and release of fish have occurred in many Japanese lakes, the changes in the plankton community described here may be widespread in these lakes.

## Introduction

To understand how anthropogenic environmental disturbances have affected lake ecosystems, firm and objective knowledge on the state of plankton communities before a disturbance is essential. Lack of long-term monitoring data has often made it difficult to gain a thorough knowledge of past communities in lakes, but it is possible to reconstruct earlier communities from plankton remains and fossil pigments of algae stored in lake sediments [1, 2]. However, these analyses have not necessarily provided a complete picture of past plankton communities because not all remains and fossil pigments stored completely and because it was difficult to identify planktonic organisms at fine taxonomic levels from their remains and fossil pigments.

Fortunately, changes in plankton communities are often characterized by replacement of particular species such as *Daphnia* that play functionally and structurally pivotal roles in aquatic food webs as keystone species [3, 4]. For better clarification of past plankton communities in lake ecosystems, several studies have used resting eggs of *Daphnia* stored in lake sediments to identify species [5], quantify individual densities [6–9], and determine genetic variations [10–15]. For example, by examining the genetic composition of *Daphnia* resting eggs in lake sediments, Brede et al. [13] showed that in Lake Constance dominant *Daphnia* species changed from native *D*. *hyalina* to invasive *D*. *galeata* when the lake eutrophied because of an increase in phosphorus loading from the watershed. Similarly, by analyzing resting eggs in lake sediments with molecular methods, Rellstab et al. [14] showed that the dominant species in some alpine lakes changed from native *D*. *longispina* to invasive *D*. *galeata* in the 1970s when the lakes were eutrophied. Both in these alpine lakes and Lake Constance, resting eggs presumably produced by hybrids between native and invasive species were found in the lake sediments dated after invasion of *D*. *galeata* [13, 14]. These findings indicated that eutrophication caused introgression or “genetic pollution” of native *Daphnia* species. Note that because resting eggs of these native and invasive species are not morphologically distinguishable, the replacement of dominant *Daphnia* species and occurrence of inter-specific hybrids cannot be revealed without genetic analyses.

**Fig 1:**
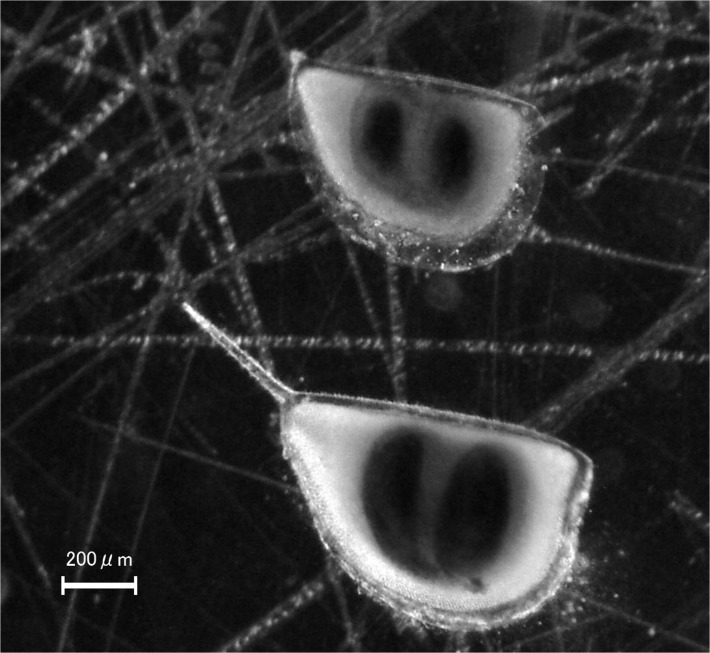
Photomicrograph as examples of ephippial carapaces of *D*. *longispina* group (above) and *D*. *pulex* (below) collected from sediment of Lake Hataya Ohnuma. Those carapaces were used for DNA extraction and were identified their species by genetic analysis.

Caution is needed, however, when reconstructing past *Daphnia* populations using resting eggs. Firstly, abundance of resting eggs stored in lake sediments may not reflect population density of *Daphnia* species in the past because the production rate of resting eggs depends on not only environmental conditions [16, 17] but also the population genetic structures [18, 19]. Thus, for estimating population density in the past, it is essential to quantify remains of planktonic individuals. Secondly, even if ephippial carapaces (Fig. 1) that are part of maternal carapaces and enclose a pair of resting eggs are stored in lake sediments, few eggs may remain in these carapaces. Indeed, it is usual that > 90% of the ephippial carapaces are empty, probably due to high hatching rates [20]. This fact implies that only a limited number of resting eggs produced in the past are available for genetic analyses. More importantly, since most individuals likely hatched from resting eggs under favorable conditions, the resting eggs remaining in the carapaces for long periods may not represent a majority of the populations [20]. Thus, genetic analyses of resting eggs remaining in lake sediments may provide a biased result. Fortunately, whether empty or not, ephippial carapaces remain for a long time in lake sediments. Recently, we have developed methods for extracting and amplifying DNA from *Daphnia* ephippial carapaces [21]. These methods enabled us to reconstruct the genetic structure of past *Daphnia* populations without a large bias.

Japan has a number of small lakes and impoundments in mountainous regions, often used to supply water for paddies and other agricultural lands. Lake Hataya Ohnuma is one such lake. As with many other lakes in these regions, the watershed is covered by forests and has few residents. Nonetheless, the lake is eutrophied. Non-native fish such as pond smelt (*Hypomesus nipponensis*) have been stocked for promoting local fishing since the 1920s, and largemouth bass (*Micropterus salmoides*) were anonymously released following a sport fishing boom in the 1980s. Little is known about when and why the lake eutrophied and how stocking and release of fish species affected plankton communities. In this study, we examined plankton remains and fossil pigments in the lake sediments of Lake Hataya Ohnuma and reconstructed the plankton community over the past 60 years to reveal ecological impacts of anthropogenic activities on small mountain lakes in Japan. In addition, we genetically analyzed ephippial carapaces of *Daphnia* in the sediments to determine if eutrophication has caused replacement of dominant *Daphnia* species as documented in some European lakes.

## Materials and Methods

### Study site

Lake Hataya Ohnuma (N 38° 14' 43.6", E 140° 12' 16.4") is located in Yamagata Citizen's Forest (a public natural park), Yamagata, Japan. The lake is about 550 m above sea level and has a surface area of 19 ha with a maximum depth of around 8 m. The watershed is about 82 ha and is covered by natural second-growth forests with broad-leaved deciduous trees and artificial plantation forests consisting of cedars and red pines (data from National Survey on the Natural Environment by the Biodiversity Center of Japan, Nature Conservation Bureau, Ministry of the Environment). According to folklore, there was a spring-fed water hole inhabited by “a dragon.” In the 1600s, local people enlarged the hole to today’s Lake Hataya Ohnuma by building an embankment to retain the water for paddies [22]. To control the supply of irrigation water for paddy fields downstream, a concrete water gate was constructed on the lakeshore in 1968 by the office of Mogami River midstream land improvement district that have jurisdiction over Lake Hataya Ohnuma. In 1968 a concrete water gate was constructed on the lakeshore to control the supply of irrigation water for paddy fields downstream. There is no inlet channel and the lake impounds spring water and also receives surface runoff from the watershed. There is no village in the watershed. However, a country inn opened on the lakeshore in 1927 and became a restaurant in 1979. In 1975 a visitor's center and public campground were constructed near the lake when a public natural park, Yamagata Citizen’s Forest, was established in the area covering the watershed of Lake Hataya Ohnuma.

Several fish species have been introduced into this lake for promoting fisheries by Sakuyazawa Fishery Cooperative, a local fishery association. In the 1920s, pond smelt (*Hyponesus nipponensis*) brought in from Lake Kasumigaura (Ibaraki, Japan) were released. These fish were released several more times prior to the 1940s in the lake. In 1985, pond smelt was released again from Lake Odawara (Aomori, Japan). Additionally, common carp, crucian carp, and eels have been released several times since 1982. In the 1980s, largemouth bass (*Micropterus salmoides*) were anonymously released without the local people’s consent and pond smelt have not been caught abundantly since then. Today, although local people have tried to remove the largemouth bass, there has been no sign of a population decrease. At the present Lake Hataya Ohnuma, concentrations of chlorophyll and total phosphorus measured by our laboratory are 5–10 μg/L and 10–20 μg/L, respectively, so nutrition condition of the lake is considered mesotrophic.

To perform this study, we had permissions to collect lake sediments and plankton of Lake Hataya Ohunma from the office of Mogami River midstream land improvement district and Sakuyazawa Fishery Cooperative that control the lake water and fisheries, respectively.

### Sediment core and dating

A total of five lake sediment cores were collected at the center of the lake using a gravity core sampler with an 11 cm diameter. Two were collected on 15 October 2008. Of these, one (A1; standard core) was used for age estimation, phosphorus measurement, phytoplankton pigment analysis and enumeration of zooplankton remains. The other (A2) was used for analyzing *Daphnia* resting eggs and ephippial carapaces. On 22 April 2010, the other three cores (B1-B3) were collected. B1 was used for additional analyses of *Daphnia* ephippial carapaces, and B2 and B3 were used for estimating magnetic susceptibilities (see below). Immediately after sampling, cores were sectioned into one cm slices and stored at 4°C in the dark. From each sediment slice, 1 cm^3^ was collected and the wet weight (WW, g/cm^3^) measured. The samples were then dried at 60°C for 48 hours to estimate dry weight (DW, g/cm^3^). Using a magnetic susceptibility meter (SM-30; ZH instruments, Brno, Czech), the samples were also used to measure magnetic susceptibilities that were indication for magnitude of sediment inflows from the watershed.

Chronology of core A1 was determined by the constant initial ^210^Pb concentration (CIC) model [23]. This model assumes a constant accumulation rate of ^210^Pb in lake sediments. Using the 22.3 year ^210^Pb half-life enabled us to estimate the lake sediment accumulation rate over the past 60 years. To confirm dating validity with ^210^Pb, we also measured ^137^Cs in the sediments, which has a peak associated with the early 1960s [24]. Activities of ^210^Pb and ^137^Cs were measured by gamma-ray spectrometry. Dried samples were sealed in standard holders to allow ^222^Rn and its short-lived daughters to equilibrate. After achieving equilibrium for at least two weeks, radioisotope concentrations were determined with a high-purity Ge-detector (GWL-120230-S, EG&G Ortec, Oak Ridge, TN, USA). The activity is expressed as excess ^210^Pb, which is defined as total ^210^Pb concentration minus the ^210^Pb produced within the sediments, assuming the supported ^210^Pb to be in equilibrium with ^214^Pb. These analyses were performed at the Center for Chronological Research, Nagoya University.

Chronologies of the other core (A2) collected in 2008 were indirectly estimated by comparing the profile of the magnetic susceptibilities with that of core A1 used in the CIC model dating. In this comparison, sediment layers showing marked peak or trough magnetic susceptibility values were used as reference years. Chronologies of other layers between the reference layers were then estimated assuming a constant sedimentation rate. We could not determine the chronology of core B1 used for genetic analysis of *Daphnia* because all the sediments in the sliced samples were sacrificed to collect ephippial carapaces. Instead, we estimated chronologies of the sediment slices in two other cores (B2 and B3) by comparing their magnetic susceptibilities with those of core A1 as above. Then, the mean chronological value at each sediment depth between these two cores was used as an approximate age to the corresponding depth of the core B1 sediment.

### Chemical analysis

Total phosphorus (TP) concentration in each layer of core A1 was measured by the molybdenum blue method [25]. Wet weights of about 0.5 g of sediment from each sectioned-layer were dissolved in 10 ml of distilled water and oxidized with persulfate at 120°C for 60 min. Supernatant was drawn off to determine total phosphorus concentration. The flux of total phosphorus per year (mg/m^2^/year) was calculated from the TP concentration (mg/g WW), the wet to dry weight ratio of the sediment (g DW/g WW) and the mass flux (g DW/m^2^/year) determined by age estimation.

### Fossil pigments and plankton remains

We extracted and quantified phytoplankton pigments in the A1 core sediments. Wet weights of about 0.5 g of sediment from each sectioned-layer were added to 10 ml of acetone and well mixed in an ultrasonic bath. The extracts were collected after centrifugation at 3,000 rpm for 10 min. These protocols were repeated until no pigment was extracted. The combined extract was evaporated to dryness under N_2_, dissolved in 3 ml diethyl ether, and washed with a 1 M NaCl aqueous solution. After evaporating the ether phase to dryness, the residue was dissolved into 200–500 ml acetone together with an internal standard, mesoporphyrin IX dimethyl ester (Sigma Chemical Co., USA), and analyzed by HPLC (Shimadzu LC-10AD) according to procedures in Kohata et al. [26] and Tsugeki et al. [27]. Chlorophyll-*a* and pheophytin-*a*, which are common in all algal taxa [28], were quantified as a surrogate of total algal biomass in the lake. In addition, taxa-specific carotenoids, fucoxanthin (derived from diatoms), echinenone (unicellular cyanobacteria), alloxanthin (cryptophytes), diatoxanthin (dinoflagellates), lutein (chlorophytes) and zeaxanthin (filamentous cyanobacteria), were quantified.

Analysis of zooplankton remains was performed according to Frey [29]. In short, wet weights of about 1.5 g of sediment from each layer of core A1 were mixed with 200 ml of water. One ml from each of these mixtures was used for microscopic observation under 100× and 400× magnification in a Sedgewick–Rafter cell. This procedure was repeated until at least 150 zooplankton fragments were counted for each sample. We identified and counted the remains of *Daphnia*, *Bosmina*, *Difflugia* spp., *Pontigulasia* sp. and *Tintinnopsis* sp. Morphologically two different *Difflugia* remains were found. These were counted separately as sp.1 and sp.2. For *Daphnia*, presence or absence of pectens on their abdominal claws was used to identify species group. We identified an abdominal claw with pectens consisting of five distinct teeth as *D*. *pulex* group and those without pectens as *D*. *longispina* group. The abundance of phytoplankton pigments (mg) and zooplankton remains (number) were converted to values per g of dry sediment. We then calculated the flux per year (mg/m^2^/year for phytoplankton, and number/m^2^/year for zooplankton) from the water column to the sediments using the sediment mass flux (g DW /m^2^/year).

In addition to these measurements, about 100 g of sediment from each sectioned-layer of core A2 was sieved with a 100-μm pore mesh. Ephippial carapaces (ECs) of *Daphnia* on the mesh were collected under a stereomicroscope. Presence or absence of eggs in the ECs was confirmed and recorded to calculate hatching rates as a proportion of empty ECs to all ECs in a layer. EC flux (number/m^2^/year) was calculated as above. Because these ECs were not preserved for further analyses, we took another set of EC samples from core B1, collected in 2010, for genetic analysis. From each layer of B1, at least 16 ECs were randomly isolated. In sediment layers containing < 16 ECs, all ECs were collected and used for extracting DNA. Although there were resting eggs presumably of rotifers and other planktonic organisms in sediments, we could not use them because of low abundance, and morphological and genetical uncertainties.

### Genetic analysis

DNA extraction from ECs was performed by the method described in Ishida et al. [21]. In short, regardless of intact of the resting eggs, ECs were individually transferred into a 0.2 ml tube containing 50 μl of alkaline lysis buffer (25 mM NaOH and 0.2 mM of disodium EDTA, pH 12) and subjected to five cycles of thermal shock consisting of 5 min. at −80°C and 20 sec. at 70°C. The samples were then vortexed followed by sonication for 1 min. using a USP-50 ultrasonic homogenizer (Shimadzu). These were incubated at 95°C for 30 min. and stored on ice for > 3 min. A further 50 μl of a neutralizing buffer (40 mM Tris-HCl, pH 5) was added to each tube. The samples were again briefly vortexed before being stored at −20°C.

PCR amplification of 12S rDNA fragments was carried out according to protocol III (using Type-it Microsatellite PCR Kit (Qiagen) and ExTaq Hot Start Version (Takara)) described in Ishida et al. [21] with the primer set composed of d5f and d7r. Four μl of the final PCR product was separated by electrophoresis in a 2.0% agarose gel to check whether a fragment was amplified. DNA sequencing reaction was performed using a BigDye Terminator v3.1 Cycle Sequencing Ready Reaction Kit (Applied Biosystems) and each PCR product purified by CleanSEQ (Agencourt Bioscience). Sequencing was performed using an ABI PRISM 3130-Avant Genetic Analyzer (Applied Biosystems). In this analysis we sequenced mitochondrial 12S rDNA and could distinguish a total of four different *Daphnia* taxa. Among these, three taxa belonged to the *Daphnia longispina* group. These were successfully separated into three haplotypes based on three different RFLP patterns [21], one type of *D*. *galeata* (G) and two types of *D*. *dentifera* (D1 and D2). We also amplified the fourth haplotype with the same primer set and identified it as *D*. *pulex*. Species identification was performed by full-length BlastN comparisons of sequences having more than 97% identity with reference GenBank *Daphnia* sequences. We counted the number of each haplotype separately in successfully amplified and sequenced samples from all layers.

### Statistical analyses

To determine chronological periods separated by major compositional changes in phytoplankton assemblages, a cluster analysis with Ward’s method [30] was performed using phytoplankton pigment (chlorophyll-*a* and pheophytin-*a*, fucoxanthin, diatoxanthin, alloxanthin, lutein, zeaxanthin and echinenone) fluxes. Before the analysis, flux data were standardized by root-mean-square deviations. To visualize direction and magnitude of temporal changes in the phytoplankton assemblages, principal component analysis (PCA) was also carried out with flux data without the standardization. These statistical analyses were performed using the R version 3.0.1 [31].

## Results

### Sediment dating

The 33-cm-long A1 core contained sediments ranging in age from 1946 in the deepest layer to 2008 at its surface according to the CIC model using ^210^Pb. Sediments from 14–15-cm and 25–26-cm-depth layers were from 1985 and 1962, respectively (S1 Table). The ^137^Cs concentration was relatively high between 17–18 cm and 27–28 cm and showed a peak at 24–25 cm. Based on the ^137^Cs profile, we assigned this layer to the early 1960s, in close agreement with the age estimated by ^210^Pb dating.

**Fig 2:**
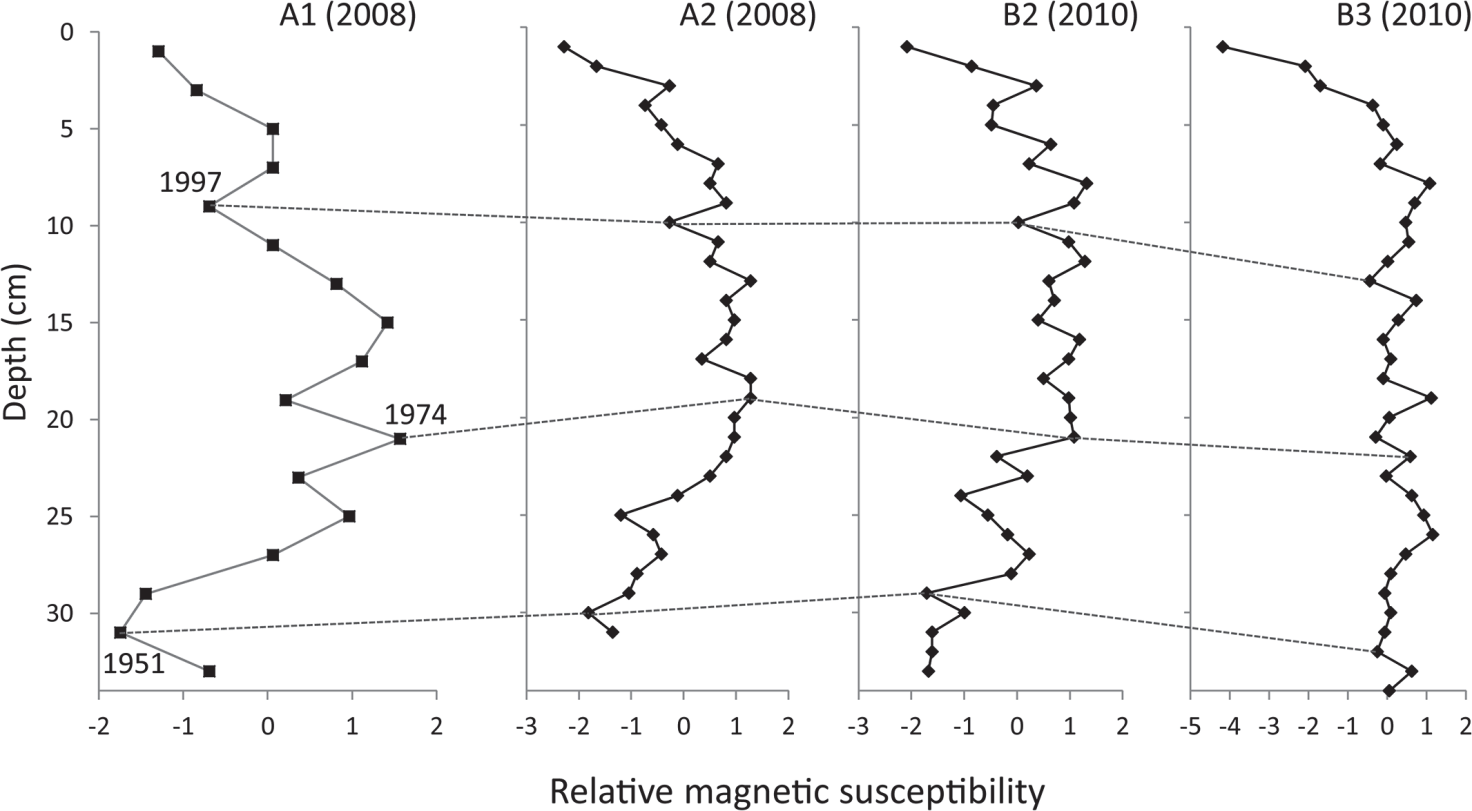
Profiles of magnetic susceptibilities in four cores of lake sediments from Lake Hataya Ohnuma. A peak and troughs of the magnetic susceptibilities in 1997 (6–7 cm), 1974 (20–21 cm) and 1951 (30–31 cm) in core A1 were used for developing chronologies of cores A2, B2 and B3. Sediment layers corresponded to the peak and toughs in core A1 are connected with dotted lines.

In all the core samples collected in this study, several peaks and troughs were found in magnetic susceptibilities (Fig. 2). Visual inspection showed that troughs at 6–7 cm (1997) and 30–31 cm (1951) and a peak at 20–21 cm (1974) in core A1 were comparable to those in other cores. Thus, these troughs and peak were used as chronological keys in dating other sediment cores. By comparing core A1 with A2, the 9–10 cm, 18–19 cm and 29–30 cm layers of A2 were dated to 1997, 1974 and 1951, respectively. Similarly, with cores B2 and B3, the 9–10 cm, 20–21 cm and 28–29 cm layers in core B2 and 12–13 cm, 21–22 cm, and 31–32 cm layers in core B3 were dated as 1997, 1974 and 1951, respectively. Ages of other layers in these cores were then estimated assuming a constant sedimentation rate between the layers dated by comparison of the magnetic susceptibilities measured in core A1. We considered that 5–6 cm or higher sediments in core A1 were mixed layers because their values of excess ^210^Pb did not show radioactive decay. Thus, the fluxes of total phosphorus, phytoplankton and zooplankton after 2000 were inaccurate and not further considered. Mean chronological values between cores B2 and B3 were used to assign ages to each layer of core B1 in support of the genetic analysis of *Daphnia* ephippial carapaces (ECs).

### Total phosphorus and fossil pigments

**Fig 3:**
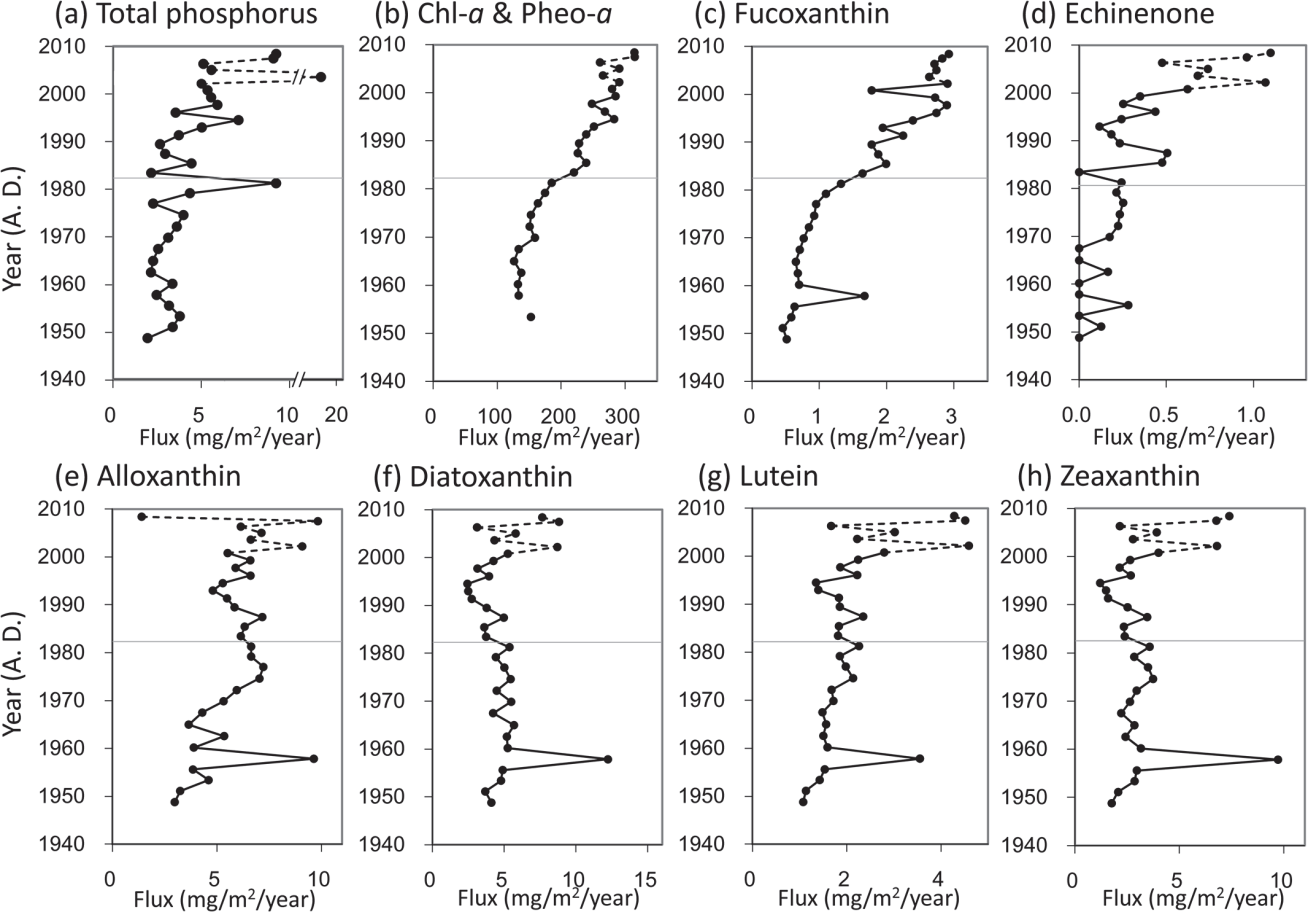
Fluxes of total phosphorus and phytoplankton pigments. Fluxes of inaccurately estimated chronological years are denoted by broken lines. Two periods (I and II) are indicated by the cluster analysis in Fig. 4 and are divided by thin line.

Analysis of core A1 showed that total phosphorus (TP) flux was at low levels from the 1950s to 1980s. After a peak in 1981 it increased gradually toward the 1990s (Fig. 3A). Flux of chlorophyll-*a* + pheophytin-*a* increased from the 1970s to 2000s (Fig. 3B). The flux in the 1990s was about two times higher than that in the 1960s. Pigment fluxes of fucoxanthin and echinenone, indicators of diatoms and unicellular cyanobacteria, respectively, generally increased from the 1970s to 2000s (Fig. 3C, D). The flux of alloxanthin increased slightly from the 1950s to 1970s but leveled off thereafter (Fig. 3E). Fluxes of diatoxanthin, lutein, and zeaxanthin had a clear peak in the 1950s, as did those of fucoxanthin and alloxanthin, but did not show large temporal changes thereafter (Fig. 3F, G, H).

**Fig 4:**
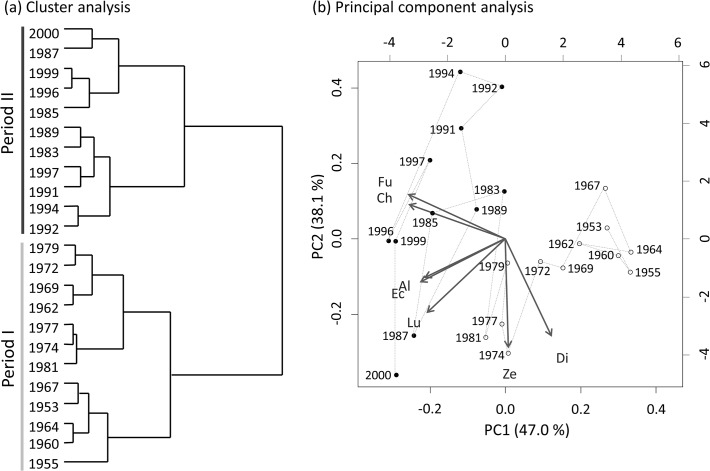
Result of the cluster analysis (a) and principal component analysis (b) using fluxes of fossil pigments. (**a)** Two distinctly divided periods are denoted by a gray line (period I) and dark gray line (period II). (**b**) Chronological years of sediment layers are denoted by open circles (period I) and closed circles (period II), and projection of vector variables of fossil pigments are denoted by gray arrows with abbreviations of pigment names, Ch: chlorophyll-*a* and pheophytin-*a*, Fu: fucoxanthin, Di: diatoxanthin, Al: alloxanthin, Lu: lutein, Ze: zeaxanthin, and Ec: echinenone.

A cluster analysis divided the stratigraphic sequence of fossil pigments into two different periods of before the 1980s (period I) and thereafter (period II) (Fig. 4A). According to principal component analysis, 85.1% of the fossil pigment flux variation was explained by the first two axes (Fig. 4B). Period I was characterized by generally low phytoplankton abundance with a relatively high abundance of diatoxanthin (dinoflagellates). In contrast, period II was characterized by high phytoplankton abundance with a dominance of fucoxanthin (Fig. 4B).

### Zooplankton remains

**Fig 5:**
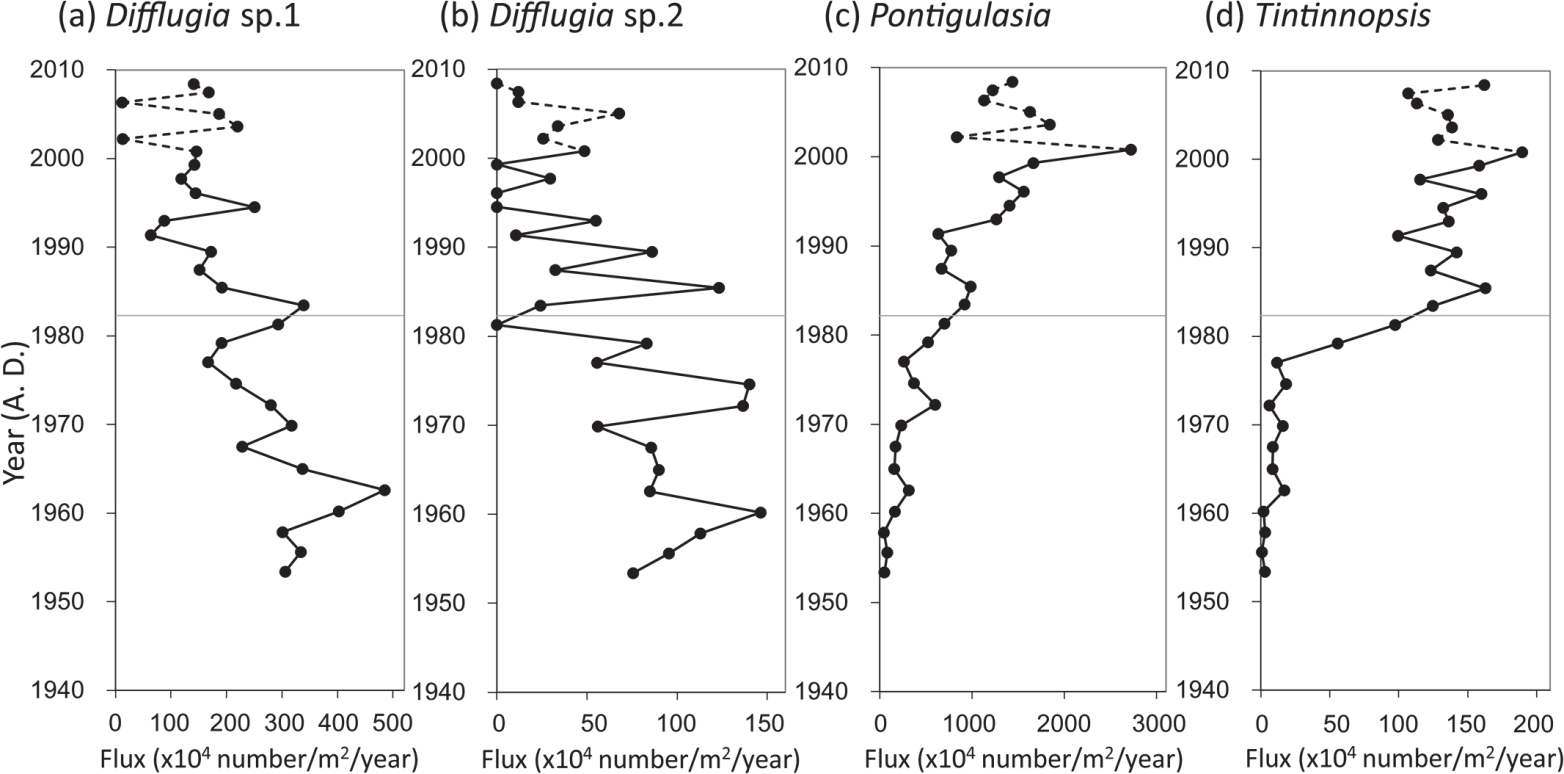
Fluxes of protozoan zooplankton remains. Fluxes of inaccurately estimated chronological years are denoted by broken lines. Two distinct periods (I and II) from the cluster analysis in Fig. 4 are divided by a thin line.

During period I, abundant remains of two morphologically different testate amoebae (*Difflugia* sp.1 and sp.2) were found in the lake sediments (Fig. 5A, B). However, fluxes of these amoebae were lower in period II. Instead, remains of another testate amoeba, *Pontigulasia*, and a ciliated protozoan, *Tintinnopsis*, that were less abundant in sediments dated before the1960s increased the fluxes gradually from the 1970s to 2000 (Fig. 5C, D).

**Fig 6:**
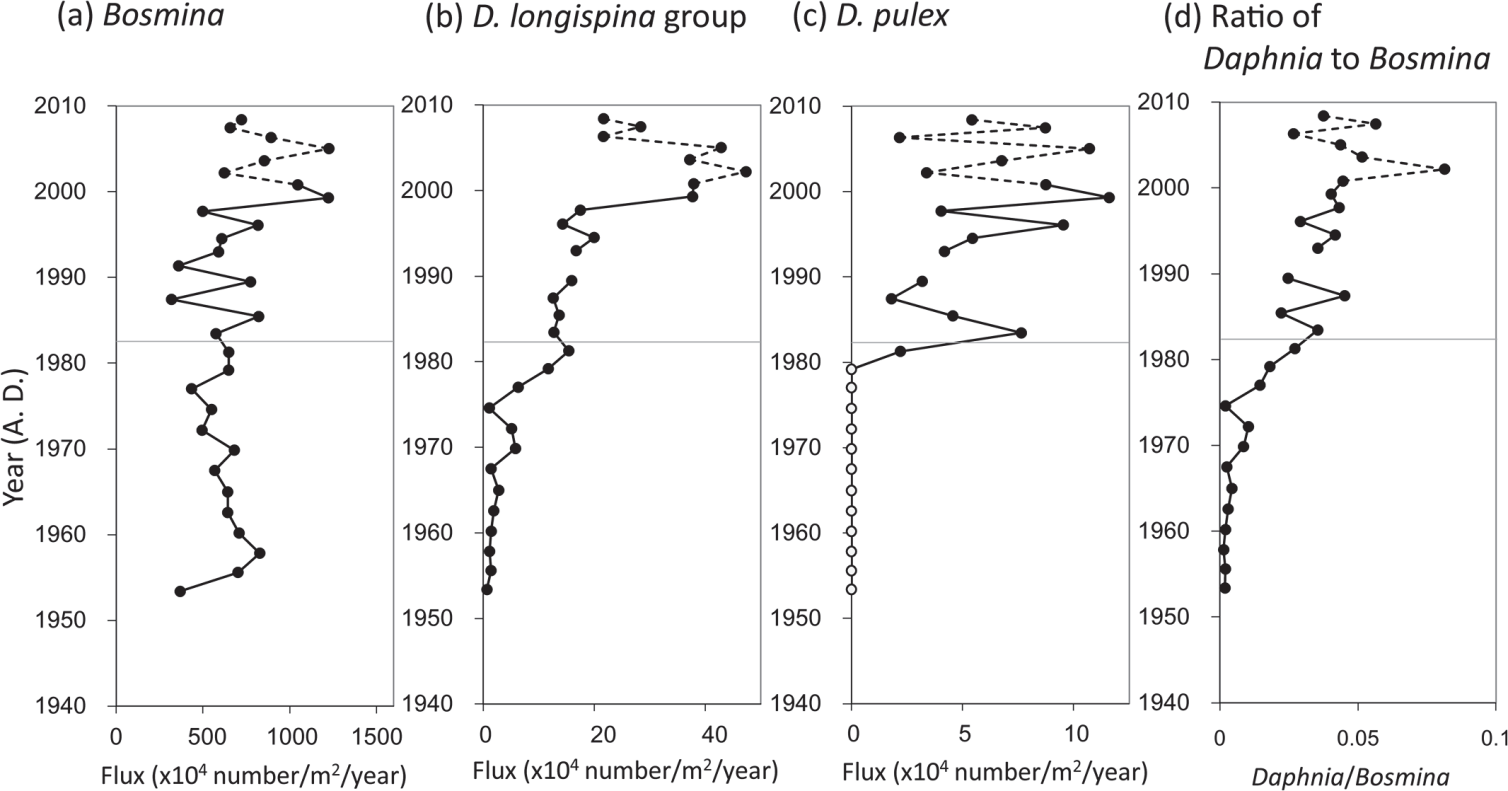
Fluxes of crustacean zooplankton remains. Fluxes of inaccurately estimated chronological years are denoted by broken lines. Two distinct periods (I and II) from the cluster analysis in Fig. 4 are divided by a thin line.

Among cladocerans, flux of *Bosmina* remains showed a relatively high level throughout the sediment layers although it fluctuated somewhat after the 1980s (Fig. 6A). Conversely, flux of *Daphnia* remains was limited before the 1970s; however, it increased dramatically during period II and reached ~50 × 10^4^ individuals/m^2^/year in 2000 (Fig. 6B, C, D). Before the 1980s, all *Daphnia* remains were of the *Daphnia longispina* group (Fig. 6B). However, we also found remains of *D*. *pulex* in the lake sediments dated after the 1980s (Fig. 6C). It appeared first in the 1981 layer and then increased slowly. The flux of this species reached > 10 × 10^4^ individuals/m^2^/year in 1999.

**Fig 7:**
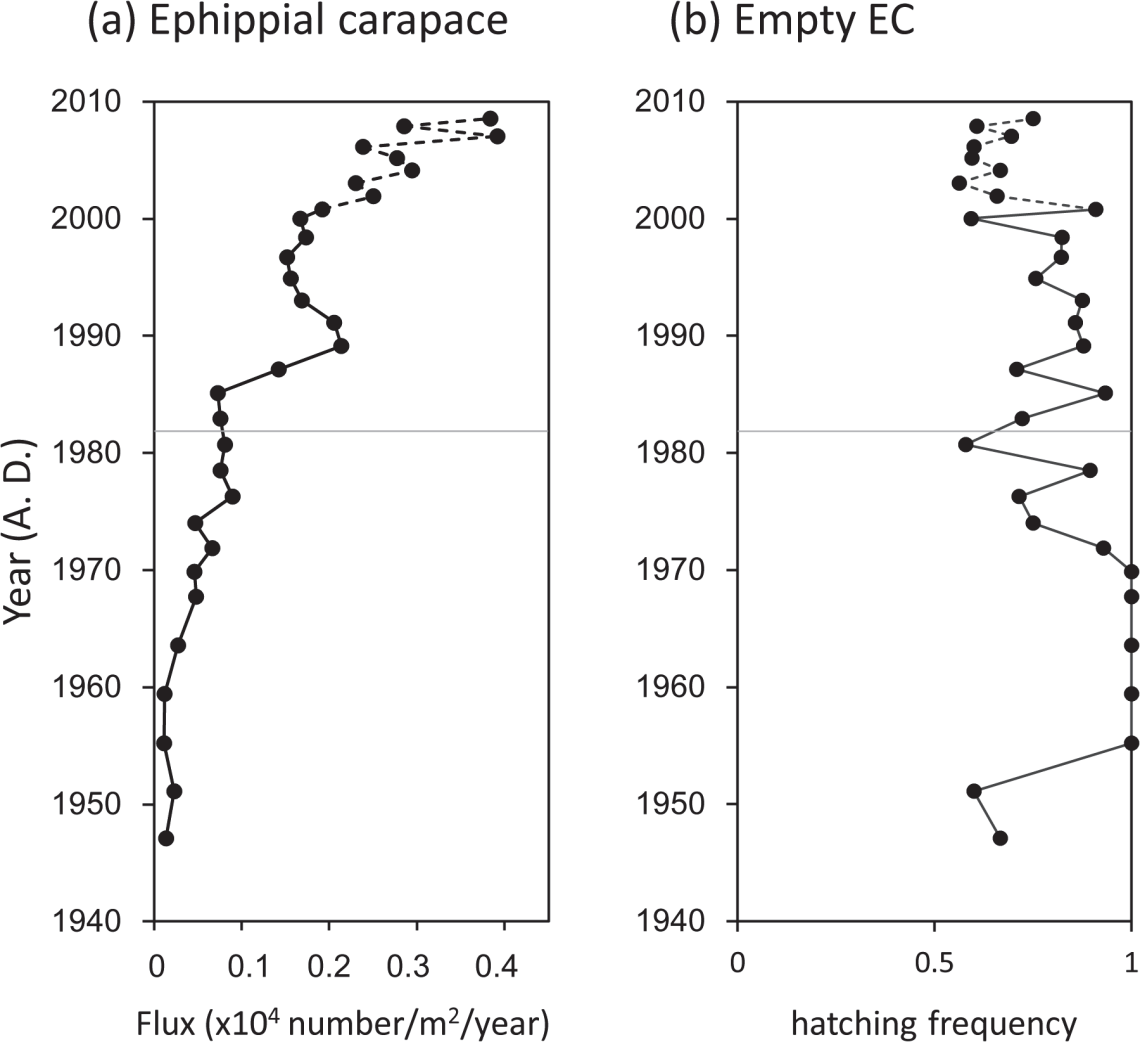
Ephippial carapace fluxes of *Daphnia* (a), and frequency of empty ephippial carapaces (b). Open circles indicate zero values. Fluxes of inaccurately estimated chronological years are denoted by broken lines. Two distinct periods (I and II) from the cluster analysis in Fig. 4 are divided by a thin line.

In parallel with *Daphnia* remains, flux of *Daphnia* ECs was < 500 /m^2^/year before 1970 but increased gradually and reached 2,000 /m^2^/year (Fig. 7A). We did not find resting eggs in the ECs in sediments dated from the mid-1950s to 1960s (Fig. 7B). Thereafter, frequency of ECs having resting eggs increased gradually with some fluctuations. However, at most it was < 30%, even in recent years.

### Genetic analysis of *Daphnia*

**Fig 8:**
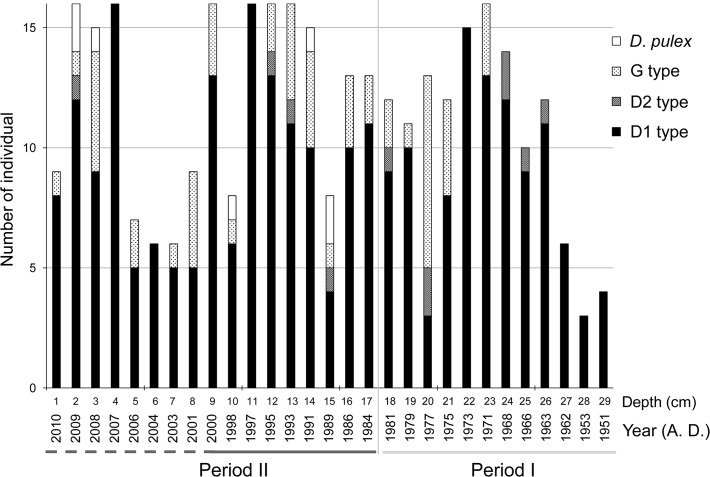
Number of ephippial carapaces with haplotypes D1, D2, G and *D*. *pulex* in different chronological layers. Total of 334 ECs were successfully analyzed. Two distinctly divided periods are denoted by a gray line (period I) and dark gray line (period II). Inaccurately estimated chronological years are shown with a broken underline.

We analyzed a total of 432 ECs stored in Lake Hataya Ohnuma sediments. Of these, 334 ECs we successfully extracted and amplified DNA and identified four haplotypes: three from the *D*. *longsipina* group (D1, D2 and G) and one from *D*. *pulex* (Fig. 8). D1 was a haplotype of *D*. *dentifera* and appeared in all layers. D2 was another haplotype of *D*. *dentifera* and was found in most but not all sediment layers between the 1960s and the present. Haplotype G was *D*. *galeata* and was first found in sediments dated from 1971 and was most abundant in sediment layers dated thereafter. The number of EC haplotype D2 was much lower than that of haplotypes D1 and G in any layer. In almost all layers, haplotype D1 accounted for > 50% of ECs in the *D*. *longispina* group. Further, concomitant with appearance of its remains (Fig. 6C), a limited number of *D*. *pulex* ECs was found in some sediment layers dated after the late 1980s.

## Discussion

This study reconstructed changes over the past 60 years in the zooplankton community of Lake Hataya Ohnuma, a small lake typical of the mountain regions of Japan. Large changes in taxonomic composition of the plankton community occurred when eutrophication progressed and when carnivorous fish were anonymously released into it. More importantly, we successfully revealed historical changes in *Daphnia* populations at the species level using genetic information from ephippial carapaces stored in the lake sediments, even though no previous monitoring data for this lake existed. Below, we first discuss changes in the plankton community in Lake Hataya Ohnuma over the past 60 years. Then, we discuss historical changes in the *Daphnia* populations in relation to eutrophication and invasion of carnivorous fish.

### Changes in the plankton community

Different from large lakes in Japan such as Lake Biwa [17, 32], Lake Kasumigaura [33, 34] and Lake Suwa [35] where large cities have developed in their watersheds, the watershed of Lake Hataya Ohnuma was protected as a local natural park and had experienced no change in land cover vegetation or land use over the past several decades. As a result, we expected that the plankton community in Lake Hataya Ohnuma was little changed for the last 60 years. However, this study showed that plankton communities, even in such a small mountainous lake, have been changed largely.

Based on fossil pigments, chronological change in the plankton community of Lake Hataya Ohnuma was divided into two periods: before 1981 (period I) and thereafter (period II) (Fig. 4). Period I was characterized by low phytoplankton abundance with low TP flux (Fig. 3). In this period, remains of *Difflugia* spp. were found in large numbers. Most *Difflugia* are benthic species [36–38] though some are often found as plankton [39]. Thus, abundant occurrence of *Difflugia* remains in the sediments suggested that the sediment surface of the lake bottom was a favorable environment for these benthic organisms in period I. However, numbers of these benthic protozoans tended to decrease since the 1970s and reached their lowest level after the1980s. Conversely, phytoplankton biomass increased gradually in the 1970s and reached high levels in period II, as evidenced by an increase in chl-*a* flux. In this period, fluxes of alloxanthin and fucoxanthin to the lake's sediments increased, suggesting that diatom and cryptomonad species became numerous. In the 1990s, flux of echinenone, a pigment from cyanobacteria, also increased. These findings suggested that although Lake Hataya Ohnuma was originally oligotrophic, eutrophication continually progressed in the 1980s. The TP flux rate after the 1990s was higher compared with that before the 1970s, which supports this suggestion. At present the lake is in a mesotrophic condition based on the TP concentration of the lake water.

Why was the lake eutrophied in the 1980s? Since the change in the trophic condition occurred only for the past 35 years, it cannot be explained by a long-term natural eutrophication. In the lake's watershed the only residents were one family of an inn that opened in 1927. As mentioned earlier, the inn was closed and opened in 1979 as a small restaurant. Thus, eutrophication progression in the 1980s cannot be attributed to activities of these few residents. In 1975 the forest area covering the watershed of Lake Hataya Ohnuma was designated as the Yamagata Citizen’s Forest and a visitor's center with a public camping area was constructed near the lake. For the first decade after the constructions of those facilities, the number of visitors was approximately 30,000 people per year on average but increased to nearly 60,000 people per year in the 1990s (Directory of Yamagata City Nature Center in 2013). Most of these visitors are school students staying for several hours to hike in the forests. Although excrement and wastewater from these visitors were deposited in sewage tanks and periodically carried out by tanker trucks to sewage plants outside the watershed, the increase in visitors may have eutrophied this lake by increasing nutrient loadings via surface runoff.

In addition to these nutrient sources in the watershed, we should consider atmospheric deposition of anthropogenically produced dust as a nutrient source contributing to the eutrophication of this small mountain lake. A number of studies have shown that increased use and emission of N and P in residential, industrial and agricultural areas have led to elevated atmospheric N and P concentrations and eutrophication progression of lakes, even in remote areas [40–43]. In Japan, atmospheric deposition of inorganic materials by locally produced dust increased from the 1960s to 1980s due to an increase in the number of vehicles and industrialization [44, 45]. Some studies showed that forests in Japan are saturated in terms of nitrogen due to atmospheric deposition over the past 40 years [46] and now deliver a substantial amount of inorganic N into ground and stream waters [47]. In recent years, an increase in nutrient deposition by atmospheric dust produced in, and transported from, continental China became a concern as a cause of eutrophication in mountain lakes in Japan [42]. In addition to the increase in nutrient loadings by forest visitors, atmospheric deposition, therefore, may have contributed to delivering nutrients into this lake and changed it from oligotorphic in period I to mesotrophic in period II.

With increasing chl-*a* flux to the lake's sediments, fluxes of *Daphnia* and *Bosmina* remains and two planktonic protozoan species, *Pontigulasia* sp. and *Tintinnopsis* sp., increased in period II (Fig. 5C, D), indicating that increased primary production caused by eutrophication was extended into zooplankton production. On the contrary, fluxes of benthic testate amoebae remains decreased as mentioned above. It is likely that the increase in algal production increased oxygen consumption on the lake bottom through decomposition of increased organic matter flux to the sediments, although anoxic layer was not yet developed at the hypolimnion even in recent years (Urabe, unpubl.). Decrease in fluxes of benthic testate amoebae remains suggests that the sediment surface of the lake became at a low oxygen condition due to eutrophication. Decrease in population density of benthic testate amoebae was also documented in Lake Biwa when eutrophication was progressed [32].

Changes in the lake bottom environment due to eutrophication were also inferred from the hatching rate of resting eggs produced by *Daphnia* species. In Lake Hataya Ohnuma flux of ephippial carapaces (ECs) of *Daphnia* resting eggs increased with that of *Daphnia* remains over the past 60 years. However, although almost 100% of ECs were empty in sediments dated before the 1980s, 10–20% of the ECs in the sediments thereafter remained intact with resting eggs. This finding shows that a substantial number of the resting eggs failed to hatch out in period II when the lake was eutrophied. Low oxygen conditions caused by eutrophication may have hampered hatching of the resting eggs produced after the 1980s.

### Changes in *Daphnia* populations

In this study we collected *Daphnia* ECs from sediment cores (A2, B1) that were not dated directly. Instead, the chronologies of these cores were indirectly estimated using vertical profiles of magnetic susceptibilities as a clue of the chronology (Fig. 2). Therefore, dates for ECs may be less precise compared with those obtained from fossil pigments and plankton remains. However, the temporal profile of EC flux was almost the same as that of *Daphnia* remains (Fig. 7). The concordance of these profiles indicates that the estimated chronology of EC flux did not deviate greatly from those of other fluxes.

To identify historical *Daphnia* species in lakes, resting eggs enveloped by ECs and stored in sediments have often been used for genetic analyses [10–15]. In Lake Hataya Ohnuma, however, more than 80% of ECs in general and almost 100% of ECs in the 1950s to 1960s did not contain resting eggs. Therefore, we could not use eggs for genetic identification of *Daphnia* species as has been occurred in other lakes [20]. Instead, we successfully extracted DNA fragments from ECs stored in the lake sediments with a novel method introduced by [21]. Then, using the RFLP method with 12S rDNA fragments, we identified three haplotypes of the *Daphnia longspina* group, and one haplotype of *D*. *pulex* from sediment ECs. The haplotypes of the *D*. *longispina* group were composed of two haplotypes of *D*. *dentifera* and one haplotype of *D*. *galeata*. Since body shape is morphologically similar and size range highly overlaps between *D*. *dentifera* and *D*. *galeata*, it is impossible to distinguish their ECs based on size and morphology. Compared with these species, *D*. *pulex* is larger in body size. Therefore, we expected that ECs of *D*. *pulex* were larger than, and thus distinguishable from, those of *D*. *dentifera* and *D*. *galeata*. However, size ranges of ECs overlapped somewhat between *D*. *pulex* and the other two species in Lake Hataya Ohnuma. Thus, genetic information was essential to identify *Daphnia* species from ECs. More importantly, genetic analysis of resting eggs stored in the sediments alone may bias reconstruction of earlier *Daphnia* populations because these eggs may be individuals that genetically failed to hatch out and thus were minor population components. Therefore, genetic analysis of ECs from the Lake Hataya Ohnuma sediments would be a more reliable approach for reconstructing past *Daphnia* populations compared with using only resting eggs.

Throughout the islands of Japan, *D*. *dentifera* is common in lakes and ponds at higher altitudes and latitudes while *D*. *galeata* occurs in lakes at lower altitudes and latitudes [48]. Lake Hataya Ohnuma is located in an overlapping area of both habitats. However, all ECs in the sediments dated before 1970 were haplotypes of *D*. *dentifera* (D1 and D2), suggesting that the *Daphnia* population in Lake Hataya Ohnuma consisted of this species alone in period I. Before the 1980s, fluxes of both *Daphnia* remains and ECs were limited. Since the lake was oligotrophic during period I, *Daphnia* abundance may have been limited by algal food supplies. However, their abundance cannot be explained by food supply alone because *Bosmina* abundantly occurred in this period compared with *Daphnia*. Although *Daphnia* are generally competitively superior to *Bosmina* [49–52], the latter occurs abundantly and dominates in lakes where predation pressure by planktivorous fish is moderately high because of their low preference as prey compared to *Daphnia* [3, 53–58]. In Lake Hataya Ohnuma, large numbers of pond smelts were stocked in period I. These fish are known to preferably prey on large zooplankton like *Daphnia* [59, 60]. Thus, in addition to a limitation by algal food supply, *Daphnia* populations were kept at low densities due to predation by pond smelts during period I, which enabled *Bosmina* to predominate over *Daphnia* in Lake Hataya Ohnuma.

In the 1971 sediment layer, *Daphnia* ECs with haplotype G were first observed, suggesting that *D*. *galeata* invaded Lake Hataya Ohnuma around 1970. In 1968 a water gate was constructed on the lake to control irrigation water supply. However, it is not clear how the water gate construction actually correlated with invasion of *D*. *galeata*. Rather, their invasion may have been related to an increase in algal production. Although the lake was notably eutrophicated in the1980s, phytoplankton had already started to increase in the 1970s. In European lakes where a native *Daphnia* species such as *D*. *hyalina* occurred, it was shown that *D*. *galeata* invaded and established populations with the advance of eutrophication [13, 14]. Thus, a similar phenomenon may have occurred at two far distant areas, Europe and Japan, owing to eutrophication as a common ecological driver. In Japan, hybrids of *D*. *dentifera* and *D*. *galeata* occurred in some overlapping areas of their habitats [48]. In a separate study, we also found that *Daphnia* individuals with haplotype D1 and haplotype G were not separated into different populations according to microsatellite analysis (Kumagai *et al*., in prep.), suggesting that invasion of *D*. *galeata* may have produced a hybrid population with *D*. *dentifera* in Lake Hataya Ohnuma. In some European lakes, invasion of *D*. *galeata* also resulted in the creation of hybrid populations with native *Daphnia* species [13, 14]. In these lakes, native *Daphnia* populations disappeared due to introgression by invading *Daphnia* species, and the resulting hybrid population became dominant [13, 14]. In Lake Hataya Ohnuma, we cannot rule out the possibility that a hybrid population originally containing haplotypes G and D invaded this lake around 1970, rather than emerging after invasion by *D*. *galeata*. It is also possible that *D*. *galeata* have invaded or existed before 1970 but may not have produced the resting eggs for a period. In any case, haplotypes D1 and G occurred abundantly over the next 40 years according to ECs stored in the lake sediments. This finding suggests that the hybrid of *D*. *galeata* and *D*. *dentifera* dominated over *D*. *dentifera* since the 1970s, although the origin of the hybrid is not clear.

In the 1980s another *Daphnia* species, *D*. *pulex*, occurred in Lake Hataya Ohnuma. Unlike *D*. *galeata*, invasion and establishment of *D*. *pulex* in this small lake cannot be explained by eutrophication. In the 1980s, largemouth bass of North American origin were anonymously and illegally introduced into the lake without the agreement of local people, as was often done in many lakes and ponds in various areas of Japan [61, 62]. Because they are piscivores, largemouth bass often reduce population densities of small fish species such as gobies, cyprinids and smelts [61, 62]. Indeed, according to local fishermen (owner of the lakeshore restaurant), abundance of pond smelt in Lake Hataya Ohnuma decreased once largemouth bass appeared. This observation suggests that introduction of this piscivore released *Daphnia* populations from predation pressures by reducing pond smelt abundance. Accordingly, *Daphnia* may have been much more abundant in period II than in period I. Supporting this inference, a number of studies have shown that anthropogenic invasions of largemouth bass caused decreases of planktivorous fishes and increases in *Daphnia* species through cascade effects [54, 55]. Since *D*. *pulex* are larger in body size than the *D*. *longispina* group, they are probably more preferable prey to planktivorous fish. Thus, illegal release of largemouth bass would have made it possible for *D*. *pulex* to invade and successfully establish their population in Lake Hataya Ohnuma.

## Conclusion

This study revealed that the plankton community of Lake Hataya Ohuma has changed over the past 60 years due to eutrophication and to the introduction of piscivorous fish, despite the fact that the watershed was protected since the mid-1970s as a natural park. More importantly, although there was no monitoring record of plankton in Lake Hataya Ohnuma, this study successfully clarified historical changes in the key zooplankton populations of *Daphnia* at the species level. To examine historical *Daphnia* populations, resting eggs stored in lake sediments have often been used in previous studies. However, if we relied on the resting eggs alone, we could not have uncovered historical population changes in Lake Hataya Ohnuma because most eggs were hatched and did not remain in the sediments dated before the 1980s. This study clearly showed that cladoceran ephipipal carapaces as well as resting eggs are useful in reconstructing past *Daphnia* populations.

In Lake Hataya Ohnuma, the *Daphnia* population was composed of *D*. *dentifera* before 1970. However, as was the case with some European lakes, *D*. *galeata* or its hybrid with *D*. *dentifera* invaded and increased the population density in the 1970s when the lake was at the onset of eutrophication. In the 1980s when largemouth bass were introduced, large *D*. *pulex* invaded and established a population. Circumstantial evidence suggests that an increase in visitors to the natural park and atmospheric deposition of dust were likely drivers causing eutrophication of Lake Hataya Ohnuma. If atmospheric deposition was the major driver, other lakes in the mountainous regions of Japan will also become eutrophied if they are not already, even if there were no notable changes in land use and land cover in the watersheds. In addition, largemouth bass have been introduced illegally in many Japanese lakes including those in mountainous areas [62]. Thus, changes in the plankton community and *Daphna* populations described here are probably not confined to Lake Hataya Ohuma but may have occurred in many other lakes in the mountains of Japan.

## Supporting Information

S1 Table 210Pb and 137Cs analytical results of core A1 for dating. (DOCX)
