# Supplementary material for: Historical Changes in the Ecosystem Condition of a Small Mountain Lake over the Past 60 Years as Revealed by Plankton Remains and Daphnia Ephippial Carapaces Stored in Lake Sediments
Source: PLoS One. 2015 Mar 10;10(3):e0119767. doi: 10.1371/journal.pone.0119767 (PMC4355284; doi:10.1371/journal.pone.0119767)
Supplement: S1 Table — (DOCX) [file pone.0119767.s001.docx]

**Table S1. ^210^Pb and ^137^Cs analytical results of core A1 for dating.**

| Depth | ^210^Pb _(exess)_ | | ^137^Cs | | ^210^Pb age (center) |
| --- | --- | --- | --- | --- | --- |
| (cm) | (Bq・g^-1^ ±s.d.) | | (Bq・g^-1^ ±s.d.) | | (year, AD) |
| 0-1 | 0.468 | ±0.032 | 0.045 | ±0.004 | 2008.40 |
| 1-2 | 0.524 | ±0.032 | 0.047 | ±0.004 | 2007.46 |
| 2-3 | 0.436 | ±0.032 | 0.045 | ±0.004 | 2006.32 |
| 3-4 | 0.498 | ±0.032 | 0.050 | ±0.004 | 2005.03 |
| 4-5 | 0.495 | ±0.032 | 0.040 | ±0.004 | 2003.61 |
| 5-6 | 0.454 | ±0.032 | 0.043 | ±0.004 | 2002.20 |
| 6-7 | 0.509 | ±0.025 | 0.048 | ±0.003 | 2000.80 |
| 7-8 | 0.420 | ±0.020 | 0.036 | ±0.002 | 1999.29 |
| 8-9 | 0.405 | ±0.020 | 0.040 | ±0.002 | 1997.71 |
| 9-10 | 0.406 | ±0.020 | 0.035 | ±0.002 | 1996.09 |
| 10-11 | 0.342 | ±0.020 | 0.037 | ±0.002 | 1994.52 |
| 11-12 | 0.380 | ±0.014 | 0.040 | ±0.002 | 1992.98 |
| 12-13 | 0.413 | ±0.023 | 0.048 | ±0.003 | 1991.35 |
| 13-14 | 0.448 | ±0.024 | 0.054 | ±0.003 | 1989.48 |
| 14-15 | 0.448 | ±0.023 | 0.051 | ±0.003 | 1987.44 |
| 15-16 | 0.339 | ±0.018 | 0.044 | ±0.002 | 1985.44 |
| 16-17 | 0.303 | ±0.019 | 0.048 | ±0.003 | 1983.44 |
| 17-18 | 0.270 | ±0.016 | 0.056 | ±0.002 | 1981.28 |
| 18-19 | 0.233 | ±0.019 | 0.052 | ±0.003 | 1979.19 |
| 19-20 | 0.228 | ±0.016 | 0.060 | ±0.002 | 1977.02 |
| 20-21 | 0.192 | ±0.015 | 0.053 | ±0.002 | 1974.60 |
| 21-22 | 0.214 | ±0.015 | 0.060 | ±0.002 | 1972.18 |
| 22-23 | 0.152 | ±0.019 | 0.054 | ±0.003 | 1969.86 |
| 23-24 | 0.167 | ±0.015 | 0.059 | ±0.002 | 1967.49 |
| 24-25 | 0.176 | ±0.019 | 0.067 | ±0.003 | 1964.98 |
| 25-26 | 0.127 | ±0.018 | 0.056 | ±0.002 | 1962.58 |
| 26-27 | 0.150 | ±0.019 | 0.058 | ±0.003 | 1960.19 |
| 27-28 | 0.149 | ±0.018 | 0.044 | ±0.002 | 1957.84 |
| 28-29 | 0.096 | ±0.013 | 0.033 | ±0.002 | 1955.60 |
| 29-30 | 0.094 | ±0.015 | 0.028 | ±0.002 | 1953.38 |
| 30-31 | 0.093 | ±0.012 | 0.018 | ±0.001 | 1951.12 |
| 31-32 | 0.110 | ±0.012 | 0.016 | ±0.001 | 1948.79 |
| 32-33 | 0.118 | ±0.017 | 0.014 | ±0.002 | 1946.50 |
|  |  |  |  |  |  |
